# Epiblast Ground State Is Controlled by Canonical Wnt/β-Catenin Signaling in the Postimplantation Mouse Embryo and Epiblast Stem Cells

**DOI:** 10.1371/journal.pone.0063378

**Published:** 2013-05-14

**Authors:** Tomoyuki Sumi, Shinya Oki, Keiko Kitajima, Chikara Meno

**Affiliations:** Graduate School of Medical Sciences, Kyushu University, Fukuoka, Japan; National University of Singapore, Singapore

## Abstract

Epiblast stem cells (EpiSCs) are primed pluripotent stem cells and can be derived from postimplantation mouse embryos. We now show that the absence of canonical Wnt/β-catenin signaling is essential for maintenance of the undifferentiated state in mouse EpiSCs and in the epiblast of mouse embryos. Attenuation of Wnt signaling with the small-molecule inhibitor XAV939 or deletion of the β-catenin gene blocked spontaneous differentiation of EpiSCs toward mesoderm and enhanced the expression of pluripotency factor genes, allowing propagation of EpiSCs as a homogeneous population. EpiSCs were efficiently established and propagated from single epiblast cells in the presence of both XAV939 and the Rho kinase (ROCK) inhibitor Y27632. Cell transplantation revealed that EpiSCs were able to contribute to primordial germ cells and descendants of all three germ layers in a host embryo, suggesting that they maintained pluripotency, even after prolonged culture with XAV939. Such an improvement in the homogeneity of pluripotency achieved with the use of a Wnt inhibitor should prove advantageous for manipulation of primed pluripotent stem cells.

## Introduction

The canonical Wnt/β-catenin signaling pathway plays pivotal roles not only in early embryogenesis but also in stem cell homeostasis and tumorigenesis [1]. The activation of Wnt signaling results in the stabilization of β-catenin through inhibition of glycogen synthase kinase 3 (GSK3), and β-catenin then translocates to the nucleus, where it serves as a coactivator for the Lef and Tcf family of DNA binding proteins in the formation of active transcriptional complexes at specific target genes [2]. Genetic studies have revealed that canonical Wnt/β-catenin signaling is essential for differentiation of the pluripotent epiblast into mesoderm in gastrulating mouse embryos [3], [4]. In contrast, the activation of Wnt/β-catenin signaling with compounds that inhibit GSK3 promotes propagation of mouse and human embryonic stem cells (ESCs) in the undifferentiated state [5], [6]. How the role of canonical Wnt signaling switches from maintenance of pluripotency in pluripotent stem cells to induction of mesoderm remains unknown, however.

Mouse epiblast stem cells (EpiSCs), which are derived from the epiblast at embryonic day (E) 5.5 to E7.5, exhibit features of pluripotency and require Nodal-Activin and fibroblast growth factor (Fgf) signaling for maintenance of this characteristic. They are therefore thought to be more closely related to human ESCs than to mouse ESCs in this regard [7], [8]. EpiSCs have little or no ability to give rise to chimeras when injected into blastocysts, suggesting that they are actually in a state of primed pluripotency, which represents a developmental state later than that of naïve mouse ESCs. A recent study showed that EpiSCs that express the E-cadherin gene (*Cdh1*) were able to contribute to embryos after blastocyst injection if they had been treated with the Rho kinase (ROCK) inhibitor Y27632 [9]. Such expression and treatment may promote cell-cell adhesion and survival, and the injected EpiSCs may be reprogrammed to a naïve pluripotent state in the blastocyst and thus rendered capable of contributing to chimeric embryos. Although EpiSCs express the core determinants of pluripotency including Oct4, Nanog, and Sox2, many pluripotency-associated transcription factors for mouse ESCs are absent or present at low levels in EpiSCs [7], [8], suggesting that these cells may possess a distinct regulatory circuitry for pluripotency. Rather than a naïve pluripotent ground state, EpiSCs appear to be in various heterogeneous states, likely reflecting their origin from a heterogeneous cell population in the epiblast at the egg-cylinder stage. They express early lineage markers for the primitive streak (PS) and mesoderm. The mixed developmental state of EpiSCs is thus considered a general hallmark of the primed state, which may restrict their pluripotency potential [10], [11]. Similar heterogeneity is also evident in human ESCs, with different levels of canonical Wnt signaling possibly underlying variability in differentiation competence [12], [13].

We have now investigated the role of canonical Wnt signaling in mouse EpiSCs with the use of small-molecule inhibitors and deletion of the β-catenin gene. Consistent with our previous observations in human ESCs [14], our data establish that canonical Wnt signaling impedes the self-renewal of EpiSCs with primed pluripotency as well as promotes mesoderm differentiation both in EpiSCs and in the postimplantation mouse embryo.

## Materials and Methods

### Mice

ROSA26-*lacZ*, *β-catenin* ^fl/fl^, and UBC-*CreER* mouse strains (The Jackson Laboratory) were maintained on the B6/129 hybrid background. Mouse embryos were collected at E6.5, with noon of the day on which the vaginal plug was detected being designated E0.5. Mice harboring the UBC-*CreER* transgene were crossed with *β-catenin* ^fl/fl^ mice. Both UBC-*CreER* and *β-catenin* ^fl/fl^ alleles were identified by PCR analysis as described previously [15]. Our study was approved by the Animal Care and Use Committee of Kyushu University (Permit Number: A24-038-1).

### EpiSC Culture

All EpiSC lines used in the present study were newly established from E6.5 epiblasts of ICR, ROSA26-*lacZ*, or *β-catenin* ^fl/fl^ mice with a modified version of a method described previously [8], [16]. In brief, isolated epiblasts were cultured and propagated on a feeder layer in EpiSC medium [Dulbecco's modified Eagle's medium (DMEM)-F12 containing 20% Knockout Serum Replacement (KSR, Invitrogen), 2 mM L-glutamine, 1 mM sodium pyruvate, 1× MEM nonessential amino acids, and 0.1 mM β-mercaptoethanol] supplemented with Activin A (10 ng/ml, Peprotech), Fgf2 (5 ng/ml, Peprotech), and XAV939 (10 µM, Sigma-Aldrich). The effects of 20 µM CHIR99021 and 0.1 µM PD0325901 on the cells were examined. For the establishment of EpiSCs derived from single cells, isolated epiblasts were incubated with trypsin-EDTA (Nakarai) at 37°C for 10 minutes and then dissociated into single cells by passage through a pipette tip. The dissociated epiblast cells were seeded on a feeder layer in four-well plates with or without 10 µM Y27632 (Wako) and 25 µM XAV939 for the indicated times. Cells were fixed with 4% paraformaldehyde in phosphate-buffered saline (PBS) for staining of alkaline phosphatase activity with nitroblue tetrazolium.

### Quantitative RT-PCR Analysis

Total RNA was extracted and purified from EpiSCs with the use of Sepasol-RNA I Super G (Nakarai) and was subjected to RT with the use of a High Capacity cDNA Reverse Transcription Kit (Applied Biosystems). Real-time PCR analysis was performed with an ABI Prism 7500 Real-time PCR system and PowerSYBR Green PCR Master Mix (Applied Biosystems). The abundance of each mRNA was normalized by the amount of 18S rRNA and calculated by the standard curve method. Details of PCR primers are available on request.

### Immunoblot, Immunofluorescence, and Immunochemical Analyses

Lysates of EpiSCs were prepared and subjected to SDS-polyacrylamide gel electrophoresis followed by immunoblot analysis as described previously [14]. For immunofluorescence analysis, cells seeded on cover glasses were fixed with 4% paraformaldehyde and permeabilized with 0.5% Triton X-100. They were then exposed to 5% bovine serum albumin (BSA) in PBS before consecutive incubations with primary antibodies and Alexa Fluor–conjugated secondary antibodies (Invitrogen). Cells were mounted on glass slides for examination with a confocal microscope (Leica TCS-SP5II). For immunochemical analysis, cells were fixed and permeabilized as for immunofluorescence analysis, incubated with 3% H_2_O_2_ for 10 minutes at room temperature, exposed to 5% BSA in PBS, and then incubated consecutively with primary antibodies and biotinylated secondary antibodies (Jackson Immunoresearch). They were then treated with ABC staining solution (Vector) and immersed in 3,3‘-diaminobenzidine solution (Vector) for detection of immunoreactivity. Primary antibodies included those to Oct3/4, to E-cadherin, and to PCNA (Santa Cruz Biotechnology); those to β-catenin (BD Biosciences); those to Sox2 (Cell Signaling); and those to Nanog (Cosmo Bio).

### Cell Transplantation

Cell transplantation was performed as described previously [17]. EpiSCs derived from ROSA26-*lacZ* embryos were treated with Y27632 (10 µM) for 1 hour and then dissociated into single cells with trypsin-EDTA. ICR embryos at E6.5 and EpiSCs were handled with manipulators (Narishige) under an inverted microscope (Zeiss). EpiSCs (10 to 20 cells) were injected between the posterior epiblast and visceral endoderm layers of E6.5 embryos in DMEM supplemented with 10% fetal bovine serum. The injected embryos were then allowed to develop in whole-embryo culture. Cells expressing *lacZ* (those derived from EpiSCs) were visualized by fixation of embryos with 1% paraformaldehyde and 0.2% glutaraldehyde in PBS for 10 minutes followed by staining with 5-bromo-4-chloro-3-indolyl-β-D-galactopyranoside (X-gal, Roche).

### Whole-embryo Culture

Whole-embryo culture was performed as previously described [18]. In brief, embryos at E6.5 were collected from pregnant ICR mice and transferred to DMEM supplemented with 10% fetal bovine serum. The embryos were then cultured under a humidified atmosphere of 5% CO_2_ at 37°C in DMEM supplemented with 75% rat serum either in four-well plates (for inhibitor experiments) or with rotation in 15-ml tubes (for cell transplantation experiments).

### In situ Hybridization

EpiSCs and embryos were fixed overnight at 4°C with 4% paraformaldehyde in PBS, dehydrated with a graded series of methanol solutions, and stored at –20°C prior to analysis. In situ hybridization was performed as described previously [18]. The *Foxd3* probe plasmid was newly generated by cloning a 1410-bp fragment of the coding region of mouse *Foxd3* amplified by PCR into pBS (Stratagene).

## Results

### Wnt/β-catenin Signaling Promotes Epiblast Differentiation

**Figure 1:**
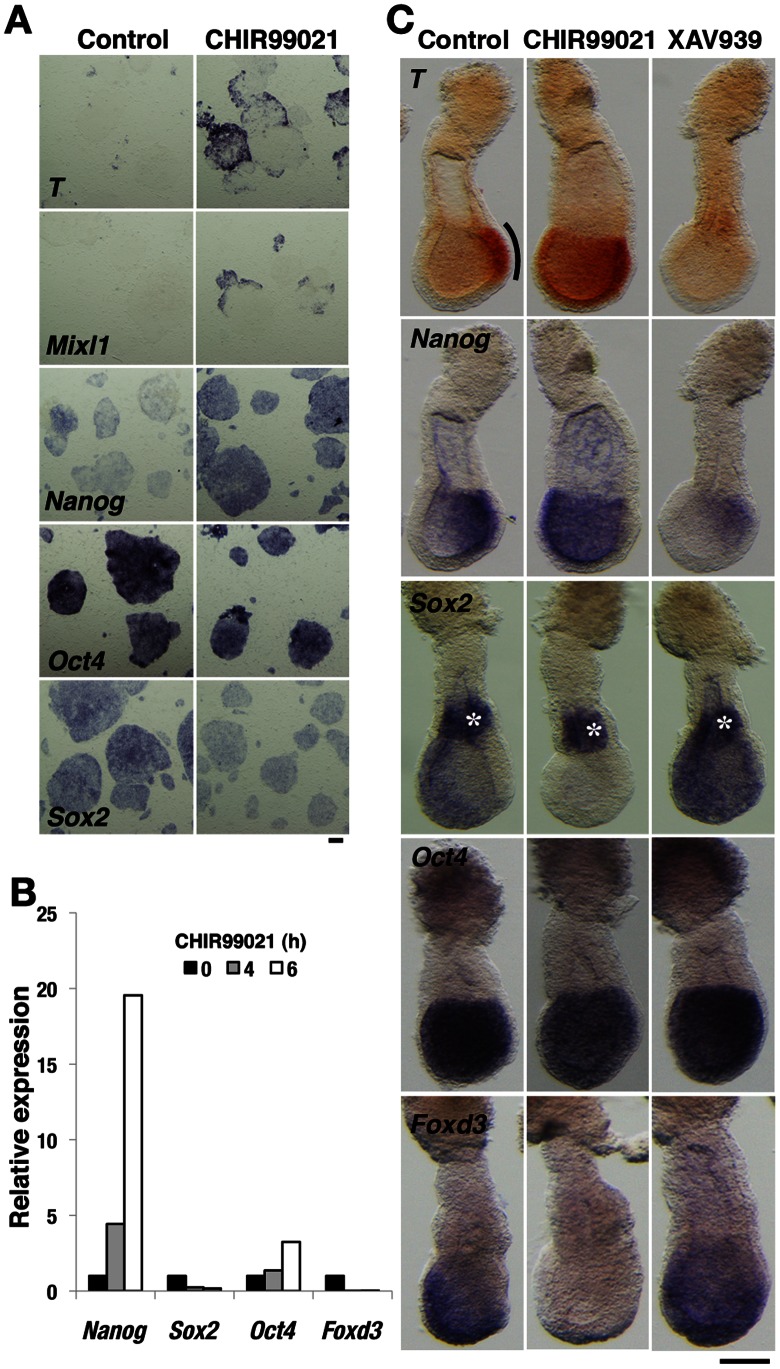
Canonical Wnt/β-catenin signaling down-regulates the pluripotency of EpiSCs and perigastrulation embryos. (**A**) In situ hybridization analysis of EpiSCs cultured without Activin and Fgf2 as well as in the absence (control) or presence of 20 µM CHIR99021 for 6 hours. Probes are indicated in each pair of panels. Scale bar, 200 µm. (**B**) Quantitative reverse transcription and polymerase chain reaction (RT-PCR) analysis of EpiSCs cultured without Activin and Fgf2 as well as in the presence of 20 µM CHIR99021 for the indicated times. The amounts of *Nanog*, *Sox2*, *Oct4*, and *Foxd3* mRNAs are shown relative to those in untreated EpiSCs. (**C**) In situ hybridization analysis of E6.5 mouse embryos cultured in the absence (control) or presence of 40 µM CHIR99021 or 100 µM XAV939 for 6 hours. Lateral views of embryos are shown with anterior to the left. The PS region is marked with a line in the *T* panels. Asterisks in the *Sox2* panels indicate expression in the extraembryonic ectoderm. Scale bar, 100 µm.

The canonical Wnt/β-catenin signaling pathway plays a major role in maintenance of pluripotent mouse and human ESCs [5], [6], but it also promotes the differentiation of human ESCs toward mesoderm [13], [14]. To investigate the role of canonical Wnt/β-catenin signaling in primed mouse EpiSCs, which closely resemble human ESCs, we first examined the effects of activating such signaling in these cells. Stimulation of the canonical Wnt signaling pathway with CHIR99021, a small-molecule inhibitor of GSK3 [19], in the absence of Activin and Fgf2 resulted in the rapid induction of *Brachyury* (*T*), *Mixl1*, and *Nanog* expression as well as in the repression of *Sox2* and *Foxd3* expression, indicative of the onset of mesoderm formation (Fig. 1A and B) [20]. To confirm the role of Wnt signaling in perigastrulation mouse embryos, we performed whole-embryo culture, which allows the direct chemical manipulation of embryos [18]. Embryos at E6.5 were cultured with small-molecule inhibitors for 6 h and then subjected to whole-mount in situ hybridization analysis. In control embryos, formation of PS-mesoderm was apparent at the posterior-proximal side of the embryo proper, as revealed by the expression of *T* and *Nanog* in this region and by the low abundance of *Sox2* and *Foxd3* transcripts in the posterior epiblast (Fig. 1C). For embryos cultured with CHIR99021, however, the region of *T* and *Nanog* expression was expanded anteriorly, whereas *Sox2* and *Foxd3* expression had disappeared from the entire epiblast, indicative of the induction of PS-mesoderm throughout the epiblast (Fig. 1C). The expression of *Sox2* in the extraembryonic ectoderm was not affected by CHIR99021, suggestive of the operation of distinct mechanisms of transcriptional regulation in the embryonic and extraembryonic ectoderm. Conversely, inhibition of Wnt signaling with XAV939, a small-molecule inhibitor of Tankyrase [21], resulted in suppression of the expression of *T* and *Nanog* as well as in maintenance of that of pluripotency factor genes such as *Oct4* (also known as *Pou5f1*), *Sox2*, and *Foxd3* in the entire epiblast (Fig. 1C). Collectively, these results supported the notion that canonical Wnt signaling induces the differentiation, rather than maintains the undifferentiated state, of EpiSCs and of the postimplantation epiblast.

### EpiSC Heterogeneity Caused by Wnt Signal Activation

**Figure 2:**
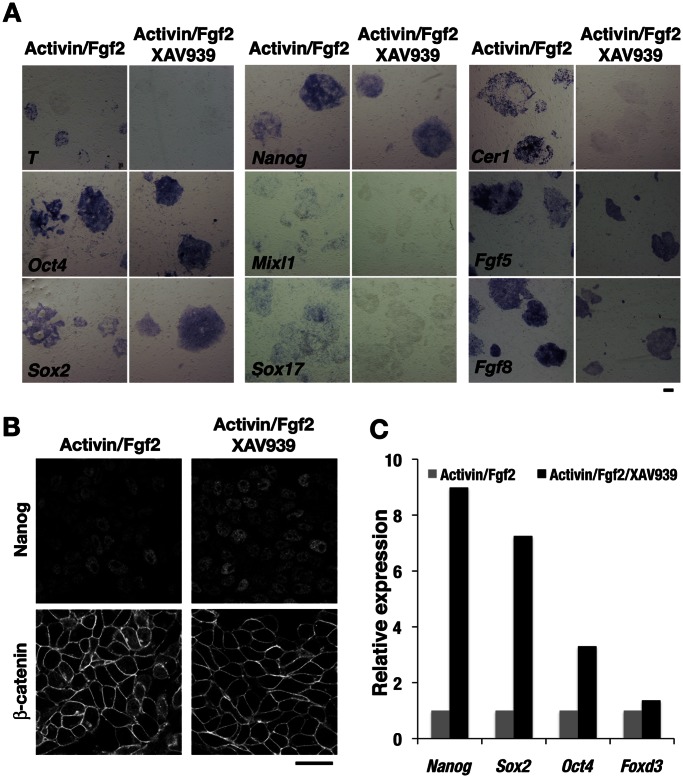
Inhibition of canonical Wnt signaling promotes the propagation of EpiSCs in the undifferentiated state. (**A**) In situ hybridization analysis of EpiSCs cultured with Activin and Fgf2 as well as in the absence or presence of 10 µM XAV939 for 2 days. Scale bar, 200 µm. (**B**) Immunofluorescence analysis of Nanog and β-catenin in EpiSCs cultured as in (A). Scale bar, 50 µm. (**C**) Quantitative RT-PCR analysis of EpiSCs cultured as in (A). The amounts of *Nanog*, *Sox2*, *Oct4*, and *Foxd3* transcripts in XAV939-treated cells are shown relative to those in untreated cells.

Mouse EpiSCs manifest various molecular features, including the expression of both pluripotency and PS-mesoderm markers [7], [8], that are consistent with the properties of the epiblast of embryos at the perigastrulation stage. At this stage, Nodal/Smad2 and Wnt/β-catenin signaling pathways in embryos regulate the induction of the PS-mesoderm [22]. We hypothesized that inhibition of these signaling pathways might allow maintenance of EpiSCs in more undifferentiated state. Consistent with previous observations [23], however, inhibition of Nodal-Activin signaling in EpiSCs with the small-molecule inhibitor SB431542 resulted in a rapid loss of the undifferentiated state and in differentiation toward the neuroectoderm lineage as revealed by the expression of *Pax6* (data not shown). In contrast, inhibition of Wnt signaling with XAV939 improved the morphology of EpiSC colonies and extinguished differentiated cells usually found in EpiSC colonies cultured with standard medium (data not shown; also see below). Given that the expression profiles of EpiSCs harboring a transgene for green fluorescent protein (GFP) under the control of the *Oct4* regulatory region were found to differ between cell subpopulations positive or negative for GFP expression [11], we examined whether PS-mesoderm marker genes were uniformly expressed among EpiSCs in the absence (control) or presence of XAV939. In situ hybridization analysis revealed a patchy and speckled pattern of *T* expression and the partial loss of *Oct4* expression within control single EpiSC colonies, indicating that EpiSCs constitute a heterogeneous population as a result of spontaneous differentiation along the mesoderm and endoderm lineages during culture in the presence of Activin and Fgf2 (Fig. 2A). In contrast, inhibition of Wnt signaling with XAV939 resulted in the uniform expression of *Oct4*, *Sox2*, and *Nanog* as well as in the disappearance of *T* expression (Fig. 2A). In addition, expression of mesoderm and endoderm markers, including *Mixl1*, *Sox17*, *and Cer1*, was completely suppressed as a result of the inhibition of Wnt signaling. The expression of *Fgf5* and *Fgf8*, which is characteristic of the epiblast state, also exhibited a more homogeneous pattern in EpiSC colonies treated with XAV939 than in control colonies (Fig. 2A). Immunofluorescence analysis showed that β-catenin was localized to the nucleus in control EpiSCs but not in the cells treated with XAV939 (Fig. 2B). The levels of transcripts derived from pluripotency factor genes such as *Nanog*, *Sox2*, and *Oct4* in XAV939-treated EpiSCs were more than three to eight times those in control cells (Fig. 2C). These data indicated that canonical Wnt signaling is a determinant of EpiSC heterogeneity as a result of its induction of mesoderm and endoderm, and that inhibition of such signaling promotes the pluripotency of EpiSCs.

### Inhibition of Wnt Signaling Promotes the Establishment and Propagation of EpiSCs in the Undifferentiated State

**Figure 3:**
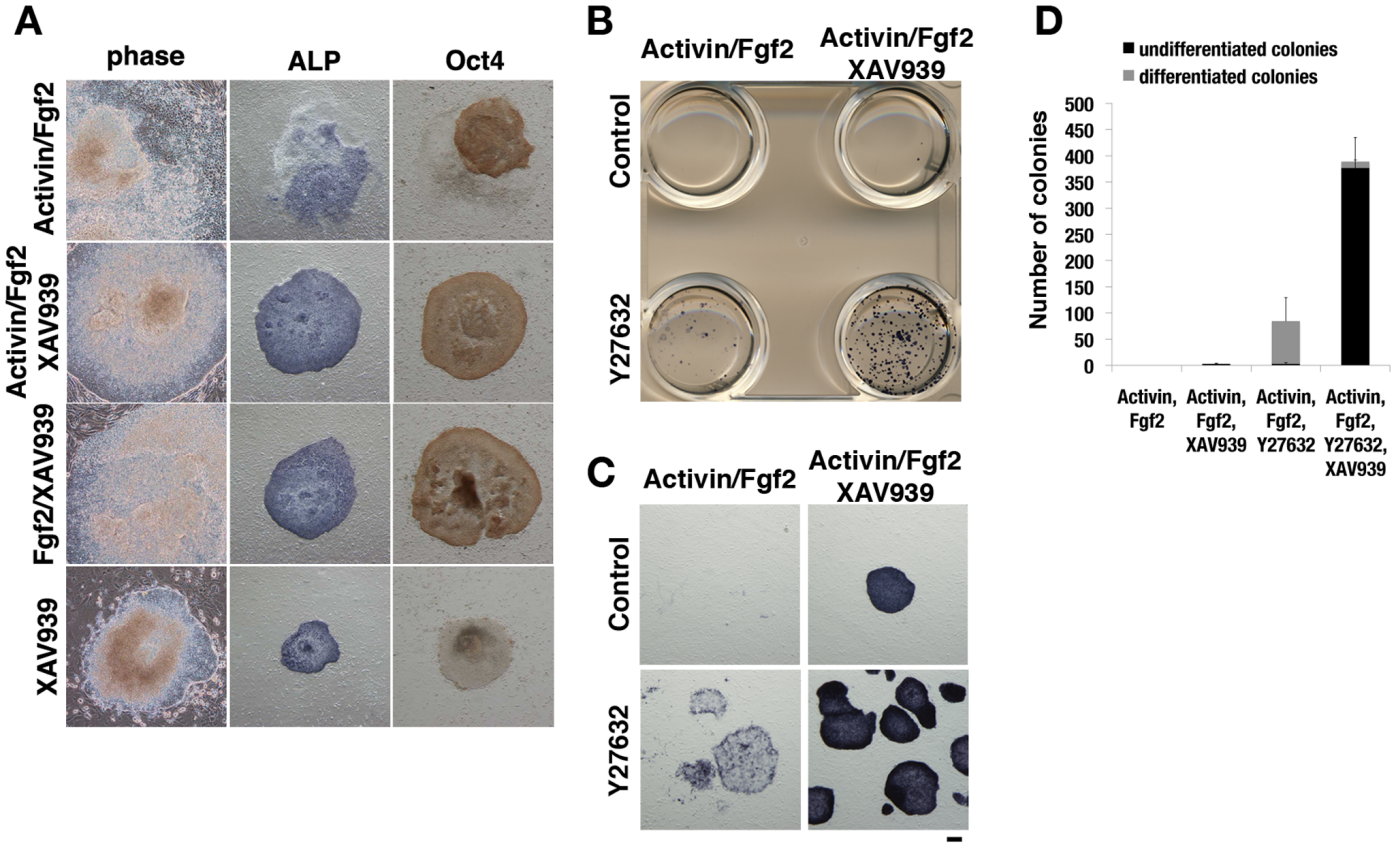
Efficient establishment of EpiSC lines from single cells. (**A**) Establishment of EpiSCs from E6.5 mouse epiblasts cultured with the indicated combinations of Activin, Fgf2, and XAV939 (25 µM) for 3 days. The cells were observed by phase-contrast microscopy, stained for alkaline phosphatase (ALP) activity (blue), or subjected to immunochemical staining for Oct4 (brown). Scale bars, 200 µm. (**B**) Culture of dissociated epiblast cells on a feeder layer with Activin and Fgf2 as well as in the absence or presence of 25 µM XAV939 or 10 µM Y27632. Epiblast cells from one embryo were seeded in each well of a four-well plate. The cells were stained for alkaline phosphatase activity after culture for 5 days. (**C**) Enlarged view of each well in (B) showing EpiSC colonies. Scale bar, 200 µm. (**D**) Number of alkaline phosphatase–positive (black bar) and –negative (gray bar) colonies for cultures similar to those in (B). Data are means ± SEM for three independent cultures per condition.

The finding of spontaneous Wnt signaling in EpiSCs prompted us to attempt the establishment of EpiSC lines comprising a more uniform cell population in the undifferentiated state through inhibition of Wnt signaling. Culture of primary epiblast explants under the standard condition with Activin and Fgf2 resulted in the formation of compact colonies of EpiSCs with a high nucleus-to-cytoplasm ratio (Fig. 3A). However, cells on the periphery of the colonies subsequently began to differentiate, giving rise to both alkaline phosphatase- and Oct4-negative cells. These differentiated cells were not observed when Wnt signaling was inhibited, although epiblast explants cultured with Fgf2 and XAV939 or with XAV939 alone could not expand continuously (Fig. 3A). Primary epiblast explants treated with the combination of Activin, Fgf2, and XAV939, however, were readily propagated for >30 passages without obvious differentiation in that the expression of mesoderm and endoderm markers remained undetectable (data not shown).

Given that EpiSCs, like human ESCs, do not survive well as single cells, we used the selective ROCK inhibitor Y27632 [24] to establish EpiSC lines derived from single cells. Dissociation of individual E6.5 epiblasts to single cells with the use of trypsin-EDTA followed by culture of the cells for 5 days under the standard condition with Activin and Fgf2 did not result in the establishment of any EpiSC lines (Fig. 3B–D). Culture in the additional presence of Y27632 yielded many large colonies, but they were almost differentiated. Although XAV939-treated epiblast cells generated few undifferentiated colonies, culture in the presence of both XAV939 and Y27632 greatly increased the number of undifferentiated colonies (Fig. 3B–D). Given that the total cell number in the epiblast at around E6.5 is thought to be in the range of 250 to 660 [25], we estimate that almost all epiblast cells produced undifferentiated stem cell colonies in the presence of these two inhibitors. Together, these results indicated that inhibition of Wnt signaling promotes EpiSC propagation in the undifferentiated state, and that Wnt signaling extinguishes the pluripotent stem cell identity of EpiSCs.

### β-Catenin is Dispensable for the Undifferentiated State of EpiSCs

**Figure 4:**
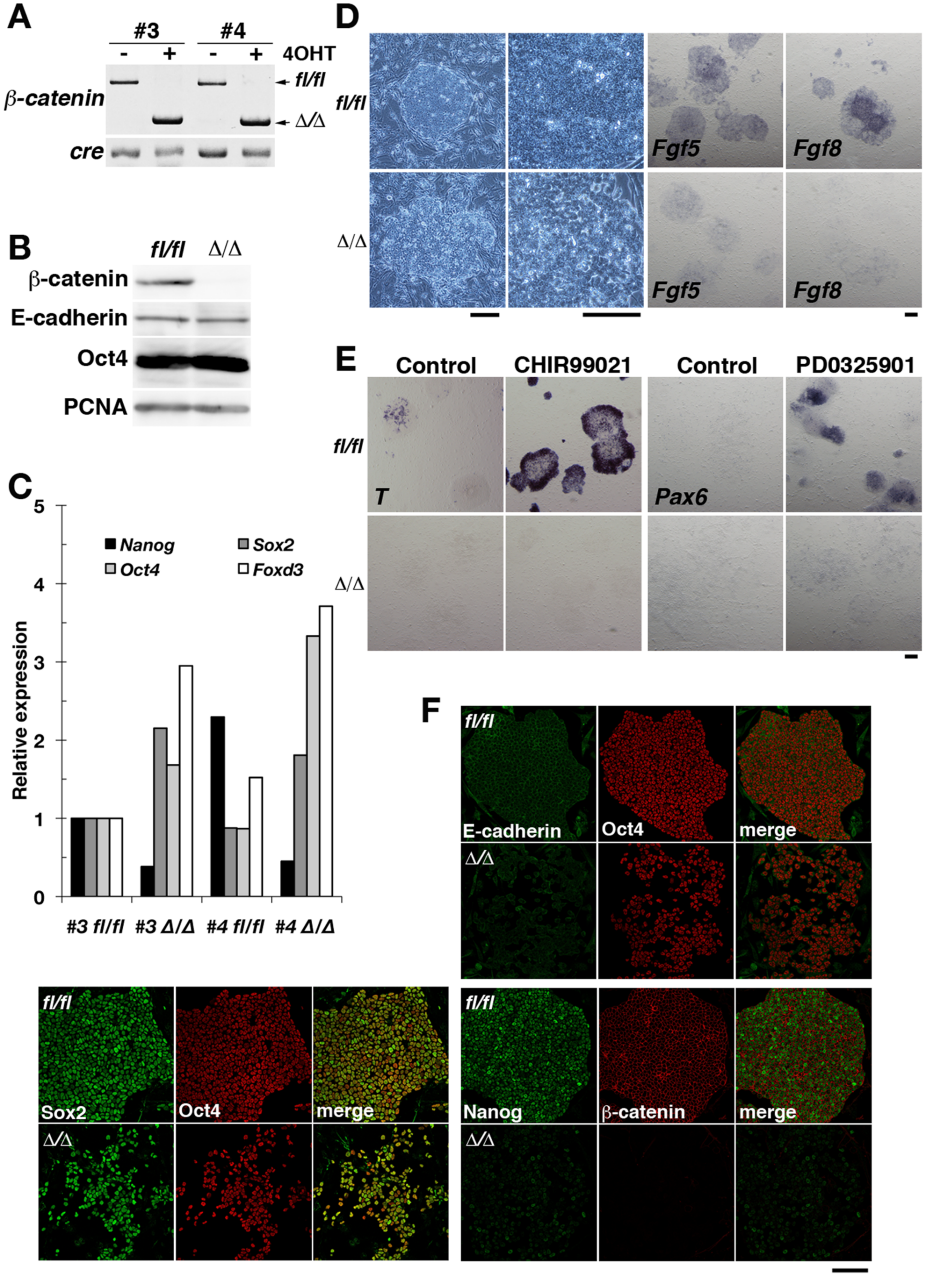
β-Catenin is dispensable for the propagation of undifferentiated EpiSCs. (**A**) Two independent EpiSC lines (#3 and #4) derived from *β-catenin* ^fl/fl^ epiblasts harboring the UBC*-CreER* transgene were incubated in the absence or presence of 100 nM tamoxifen (4OHT) for four days, after which *β-catenin* and *Cre* genotype was determined by PCR analysis. The positions of amplification products corresponding to the floxed (fl/fl) and null (Δ/Δ) alleles of *β-catenin* as well as to the *Cre* transgene are indicated. (**B**) Immunoblot analysis of β-catenin, E-cadherin, Oct4, and proliferating cell nuclear antigen (PCNA) in EpiSCs of line #3 in (A). (**C**) Quantitative RT-PCR analysis of the indicated transcripts in EpiSC lines as in (A). The amounts of *Nanog*, *Sox2*, *Oct4*, and *Foxd3* transcripts are shown relative to those in *β-catenin* ^fl/fl^ cells of line #3. (**D**) Phase-contrast images and in situ hybridization analysis of the epiblast markers *Fgf5* and *Fgf8* in *β-catenin* ^fl/fl^ or *β-catenin* ^Δ/Δ^ EpiSC colonies of line #3. Scale bars, 200 µm. (**E**) In situ hybridization analysis of *T* and *Pax6* expression in *β-catenin* ^fl/fl^ or *β-catenin* ^Δ/Δ^ EpiSC colonies of line #3 cultured with or without 20 µM CHIR99021 for 6 h or 100 nM PD0325901 for 2 days. Scale bar, 200 µm. (**F**) Immunofluorescence analysis of E-cadherin, Oct4, Sox2, Nanog, and β-catenin in *β-catenin* ^fl/fl^ or *β-catenin* ^Δ/Δ^ EpiSC colonies of line #3. Scale bar, 200 µm.

To confirm further this notion, we took advantage of the availability of β-catenin–deficient EpiSCs and examined whether it was possible to maintain these cells in the undifferentiated state. EpiSC lines were established from E6.5 epiblasts of mice that were homozygous for a floxed allele of the β-catenin gene (*β-catenin* ^fl/fl^) and which also harbored a transgene (UBC*-CreER*) for a tamoxifen-activatable form of Cre recombinase. The cells were rendered null for *β*-*catenin* (*β*-*catenin* ^Δ/Δ^) by treatment with tamoxifen. Two independent cell lines (designated #3 and #4) were established, one of which (#3) was subjected to further characterization (Fig. 4). Genotyping by PCR and immunoblot analyses confirmed the complete elimination of both the β-catenin gene and protein in *β*-*catenin* ^fl/fl^ cells in response to tamoxifen treatment (Fig. 4A and B). Whereas *β*-*catenin* ^fl/fl^ EpiSCs produced compact colonies typical of EpiSCs, the *β*-*catenin* ^Δ/Δ^ cells were loosely associated with each other (Fig. 4D), with the result that they were readily dissociated by treatment with collagenase (data not shown). In *β*-*catenin* ^fl/fl^ cells, E-cadherin accumulated at sites of cell-cell adhesion, where adherens junctions formed (Fig. 4F). In contrast, E-cadherin was diffusely distributed in *β*-*catenin* ^Δ/Δ^ cells, suggestive of an altered state of cell-cell adhesion (Fig. 4F) [26]. The abundance or distribution of Oct4 and Sox2 did not appear to differ between *β*-*catenin* ^fl/fl^ and *β*-*catenin* ^Δ/Δ^ cells, although the levels of *Oct4*, *Sox2*, and *Foxd3* transcripts in *β*-*catenin* ^Δ/Δ^ cells were two- to threefold those in *β*-*catenin* ^fl/fl^ cells (Fig. 4B, C and F). In addition, the expression of *T* was not observed in *β*-*catenin* ^Δ/Δ^ cells (Fig. 4E), suggesting that EpiSCs lacking β-catenin preserve the undifferentiated state. The levels of both Nanog protein and mRNA were down-regulated in *β-catenin* ^Δ/Δ^ cells compared with those in *β-catenin* ^fl/fl^ cells, however, and expression of the epiblast markers *Fgf5* and *Fgf8* was also diminished in the *β*-*catenin* ^Δ/Δ^ cells (Fig. 4C, D and F), suggesting that the *Fgf* genes are downstream targets of canonical Wnt signaling [27]. These changes in *β*-*catenin* ^Δ/Δ^ cells were not likely attributable to differentiation because *Oct4* and *Sox2* were stably expressed. To test whether the ablation of β-catenin in EpiSCs affects differentiation potential, we examined the effects of inducers of differentiation toward mesoderm or neuroectoderm. Consistent with previous results showing that *T* is a target of canonical Wnt signaling [3], [4], *β*-*catenin* ^Δ/Δ^ cells failed to up-regulate *T* expression even when cultured in the presence of CHIR99021 (Fig. 4E). Furthermore, whereas the MEK inhibitor PD0325901 [28], [29] resulted in the formation of neuroectodermal cells positive for *Pax6* expression by *β*-*catenin* ^fl/fl^ cells, it had no such effect in *β*-*catenin* ^Δ/Δ^ cells (Fig. 4E). Cell-cell adhesion dependent on β-catenin has been shown to be necessary for neural differentiation of mouse ESCs [30], [31]. Together, our data indicated that cell adhesion and transcriptional regulation mediated by β-catenin are not required for maintenance of self-renewal in EpiSCs, whereas β-catenin is required for differentiation toward mesoderm and neuroectoderm.

### Transplantation of EpiSCs Generates Chimeric Embryos

**Figure 5:**
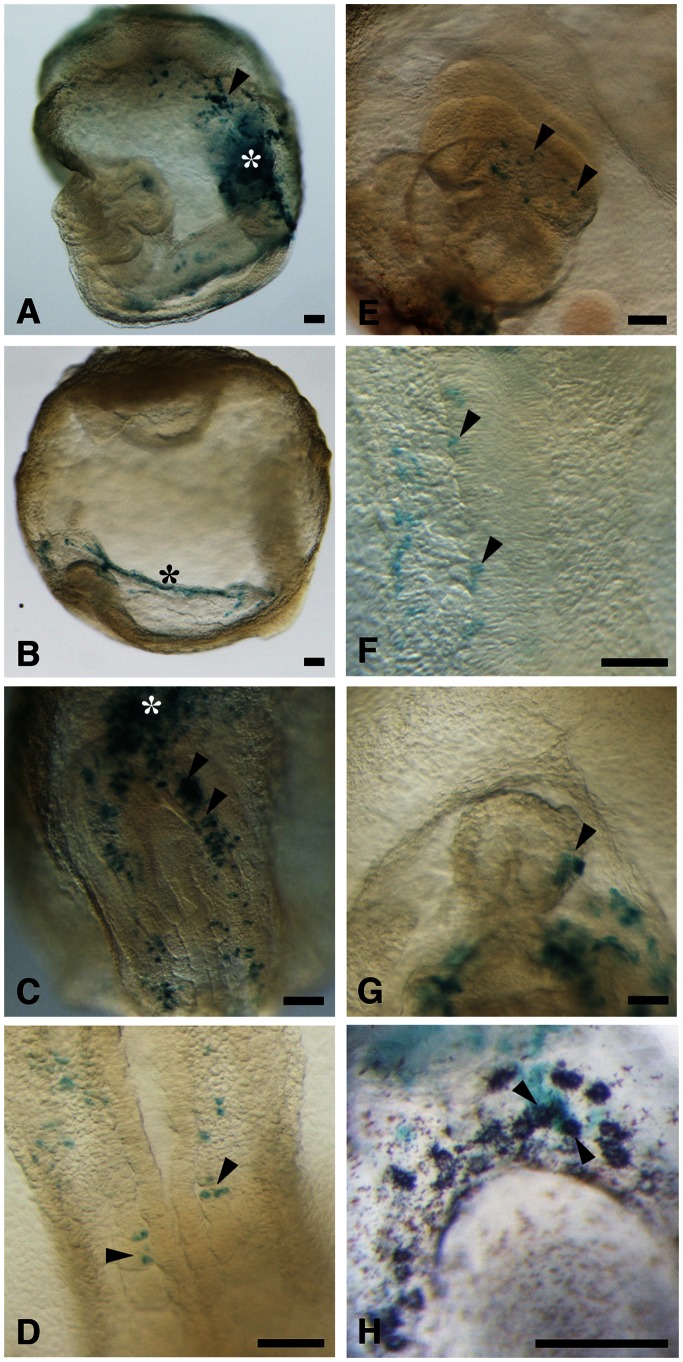
EpiSCs cultured with XAV939 contribute to chimeric embryos. Mouse embryos at E6.5 were injected with EpiSCs that harbor a *lacZ* transgene (ROSA26-*lacZ*) and which had been cultured in the presence of 10 µM XAV939 for at least 10 passages and exposed to 10 µM Y27632 for 1 hours before dissociation into single cells. The embryos were cultured until the early somite stage and then subjected to X-gal staining (green). The embryo in (H) was also stained for alkaline phosphatase activity (purple). LacZ-positive descendants of EpiSCs were detected in the allantoic bud [asterisks in (A) and (C)], yolk sac [arrowhead in (A)], amnion [asterisk in (B)], hindgut endoderm [arrowheads in (C)], somitic mesoderm [arrowheads in (D)], surface ectoderm [arrowheads in (E)], neural plate [arrowheads in (F)], myocardium [arrowhead in (G)], and migrating PGCs [arrowheads in (H)]. Embryos are oriented with anterior to the left (A, B, and E), posterior to the top (C, D, and H), or anterior to the top (F and G). A distal view of somites (D), enlarged view of the headfold (E), dorsal view of the neural plate (F), and ventral view of the heart primordium (G) are shown. Scale bars, 100 µm.

Finally, we examined the ability of XAV939-treated EpiSCs to differentiate in the developing mouse embryo. Given that EpiSCs were previously found not to contribute to chimeras after blastocyst injection [7], [8], we performed cell transplantation in the gastrulating mouse embryo [32], [33]. EpiSCs expressing β-galactosidase (derived from ROSA26-*lacZ* embryos) were established with XAV939, treated with Y27632, and transplanted into the space between the epiblast and visceral endoderm of E6.5 embryos, which were then cultured for 2 days to allow development to the four- to six-somite stage. The embryos (*n* = 18) were then stained with the β-galactosidase substrate X-gal and examined for the distribution of EpiSC-derived cells. EpiSCs were found to contribute preferentially to the extraembryonic mesoderm including the allantoic bud (*n* = 17/18), yolk sac (*n* = 10/18), and amnion (*n* = 7/18) (Fig. 5A–C). Some cells also contributed to derivatives of endoderm (*n* = 11/18), mesoderm (*n* = 7/18), and ectoderm (*n* = 3/18) (Fig. 5C–G). Teratoma formation, a defining trait of pluripotency, was not observed in any of the EpiSC-grafted embryos. Given that primordial germ cells (PGCs) migrate from the extraembryonic mesoderm to the hindgut endoderm beginning around E7.5 [34], [35], we examined whether the transplanted EpiSCs were able to contribute to PGCs in the hindgut. Migrating PGCs at the base of the allantois and hindgut endoderm can be distinguished from other embryonic cells by their expression of the tissue-nonspecific form of alkaline phosphatase (TNAP) at the cell membrane [34], [35]. Double-staining for alkaline phosphatase and β-galactosidase activities revealed that some alkaline phosphatase–positive PGCs also exhibited β-galactosidase activity (*n* = 3/8 embryos) (Fig. 5H), indicative of the differentiation of EpiSCs into PGCs in vivo. Given that the EpiSCs were introduced into the space between the epiblast and visceral endoderm, and not into the epiblast layer, these cells might directly differentiate into various cell types without the gastrulation process in the epiblast. Together, these results indicated that EpiSCs have the developmental potential to form derivatives of all three embryonic germ layers as well as PGCs, and that they are able to contribute normally to the developing mouse embryo even after prolonged culture in the presence of an inhibitor of Wnt signaling.

## Discussion

We have shown that the canonical Wnt/β-catenin signaling pathway attenuates the undifferentiated state of EpiSCs and the epiblast of postimplantation mouse embryos. Importantly, expression of PS-mesoderm markers, which has been considered characteristic of EpiSCs, was found to be heterogeneous in these cells and to be extinguished by inhibition of Wnt signaling. These data indicate that the expression of PS-mesoderm markers is a consequence of spontaneous differentiation rather than a general hallmark of the primed pluripotent state. In mouse embryos, *Wnt3* is expressed in the posterior epiblast for induction of the PS, where mesoderm cells are generated [3]. This fact suggests that EpiSCs in which canonical Wnt signaling has not been activated represent the ground state of primed pluripotency of the postimplantation epiblast. The heterogeneity caused by canonical Wnt signaling may be responsible in large part for the observed inefficiency in the direction of primed pluripotent stem cells toward specific cell types [10], [12], [36]. The improvement in primed pluripotency observed in response to XAV939 treatment in the present study may also prove to be applicable not only to manipulation of human ESCs and induced pluripotent stem cells (iPSCs) of uniform quality but also to increasing the efficiency of somatic cell reprogramming in the establishment of human iPSCs. In mouse ESCs, the canonical Wnt signaling pathway functions to maintain pluripotency [5], [6]. On the other hand, β-catenin has been found to be dispensable for the pluripotency of these cells [30], [37]. We have now shown that canonical Wnt signaling induces the differentiation of EpiSCs toward mesoderm, consistent with its function in mouse gastrulation [3], [4]. In human ESCs, the canonical Wnt signaling pathway induces differentiation rather than maintains pluripotency [13], [14]. The mechanisms by which canonical Wnt signaling promotes pluripotency in mouse ESCs and differentiation in human ESCs and mouse EpiSCs remain unclear. The modes of LIF/Stat3, BMP, Activin-Nodal, and Fgf/MAPK signaling have been found to differ between naïve and primed pluripotent cells [38]. These signaling pathways influence the functions of core transcription factors and other pluripotency-associated factors. The responses of naïve and primed pluripotent cells to canonical Wnt signaling may thus differ depending on the status of other signaling pathways that serve to maintain the core circuit of pluripotency.

Although several downstream targets of canonical Wnt/β-catenin signaling have been identified [39], [40], the target genes required for pluripotency and PS-mesoderm differentiation remain to be identified. Recent studies have shown that *Esrrb* is a pivotal target of β-catenin/Tcf3 as well as of Nanog in regulation of the naïve pluripotent state [41], [42], [43]. However, it seems likely that the β-catenin/Tcf3/Esrrb pathway does not function in the primed pluripotent state, given that inhibition of GSK3 induces mesoderm differentiation (Fig. 1) rather than the expression of *Esrrb* in EpiSCs [42]. We found that *Nanog*, *Fgf5*, and *Fgf8*, all of which are downstream target genes of Nodal signaling [23], [43], are also regulated by β-catenin in EpiSCs. The expression of these genes was thus markedly diminished in *β-catenin* ^Δ/Δ^ cells even in the presence of Activin and Fgf2. The nuclear translocation of Smad2/3 did not differ between *β-catenin* ^fl/fl^ and *β-catenin* ^Δ/Δ^ cells (data not shown), indicating that Nodal signaling was not impaired by the loss of β-catenin. It is thus possible that β-catenin acts directly or cooperatively with Nodal signaling to regulate the expression of these genes in EpiSCs and the postimplantation epiblast. Given that XAV939 treatment conferred uniform expression of *Nanog*, *Fgf5*, and *Fgf8* in EpiSCs, a Wnt-independent function of β-catenin may be required for Nodal signaling to induce the expression of these genes.

Evidence suggests that Nanog prevents neuroectoderm differentiation and supports the pluripotent state in EpiSCs and human ESCs [23]. Fgf signaling also inhibits neural commitment in EpiSCs and human ESCs [28], [44]. We were not able to induce expression of the neuroectoderm marker *Pax6* in *β-catenin* ^Δ/Δ^ EpiSCs by inhibiting Fgf/MAPK signaling, even though *Nanog* expression was down-regulated in these cells. These results suggest that β-catenin is essential for neural induction, and that Nanog is dispensable for inhibition of neural differentiation in *β-catenin* ^Δ/Δ^ cells. Consistent with this notion, Nanog was recently shown not to be required for establishment and maintenance of both naïve and primed pluripotency [41], [45], [46]. The failure to induce neuroectoderm in *β-catenin* ^Δ/Δ^ EpiSCs is seemingly inconsistent with the fact that the neuroectoderm is formed in the anterior epiblast, which is devoid of canonical Wnt signaling. A function of β-catenin other than that in canonical Wnt signaling, such as in cell adhesion, may be required for the induction of neuroectoderm.

We also found that the canonical Wnt/β-catenin signaling pathway negatively regulates *Sox2* and *Foxd3* expression in EpiSCs and gastrulating embryos. *Sox2* and *Foxd3* function in the maintenance of pluripotency in epiblast cells, with corresponding mutant embryos having been shown to die after implantation as a result of a loss of epiblast cells [47], [48]. Both genes also contribute to neural fate commitment [49], [50]. Consistent with this latter function, the expression of *Sox2* and *Foxd3* persists in the anterior epiblast during neuroectoderm differentiation, whereas the expression of these genes in the posterior epiblast is down-regulated during PS formation (Fig. 1C). Our results with embryo culture indicate that canonical Wnt signaling in the posterior epiblast attenuates the expression of *Sox2* and *Foxd3*. Expression of *Sox2* was previously shown to be increased in the posterior region of embryos lacking β-catenin [51]. Although it has not been examined, it is possible that Wnt3, a canonical Wnt ligand expressed at the perigastrulation stage, is responsible for this down-regulation of *Sox2* and *Foxd3* [3]. Recent studies have shown that Oct4 and Nanog suppress the neuroectodermal fate and promote mesodermal differentiation, whereas Sox2 suppresses mesodermal fate, during the differentiation of mouse ESCs [23], [52], [53]. Given that pluripotent factors participate in the differentiation of pluripotent stem cells, we therefore propose that germ layer specification during gastrulation may depend on transcriptional regulation of pluripotent factors by Wnt/β-catenin signaling.

In summary, our data show that inhibition of canonical Wnt signaling confers a uniform pluripotent ground state on EpiSCs. Given the functional and molecular differences detected among human ESC lines as well as among human iPSC lines [36], [54], treatment with an inhibitor of canonical Wnt signaling might allow the isolation of primed pluripotent human ESCs and iPSCs of uniform and high quality. Further studies are warranted to identify the target genes of canonical Wnt signaling and to reveal the mechanisms by which such signaling elicits different responses in naïve and primed pluripotent stem cells.
